# Habitual caffeine intake, genetics and cognitive performance

**DOI:** 10.1177/02698811241303601

**Published:** 2024-12-08

**Authors:** Angeliki Kapellou, Leta Pilic, Yiannis Mavrommatis

**Affiliations:** Faculty of Sport, Allied Health and Performance Science, St Mary’s University Twickenham, London, UK

**Keywords:** Attention, coffee, cognition, memory, polymorphism

## Abstract

**Background:**

Research on caffeine and cognitive performance remains controversial. Variations in genes associated with caffeine metabolism and response such as *CYP1A2, AHR* and *ADORA2A* may account for variable findings.

**Aim:**

To investigate caffeine × gene interactions on cognitive performance in all key domains of cognition in healthy individuals.

**Methods:**

Participants completed a lifestyle and food frequency questionnaire and a cognitive test battery including validated tasks to assess the domains of social cognition, memory, attention and executive function. Genotyping was performed for *AHR* rs6968554, *CYP1A2* rs2472297, *ADORA2A* rs5751876, *ADA* rs73598374 and *APOE* rs429358 and rs7412.

**Results:**

Significant gene × caffeine interactions were observed for the domains of social cognition, (*F*_2, 123_ = 5.848, *p* = 0.004) and executive function (*F*_2, 109_ = 3.690, *p* = 0.028). ‘Slow’ metabolisers had a higher performance in social cognition compared with ‘fast’ metabolisers among high-caffeine consumers (*p* = 0.004), while ‘fast’ metabolisers had a higher performance in executive function compared with ‘slow’ metabolisers among moderate caffeine consumers (*p* = 0.002).

**Conclusions:**

The present findings suggest an association between genetic caffeine metabolism, habitual caffeine intake and cognitive function in the domains of social cognition and executive function. More research in naturalistic environments using larger cohorts is needed to confirm these findings to add to our understanding of how habitual caffeine may influence cognitive function based on individual genotype.

## Introduction

The interest in the effects of various agents on cognitive function has grown remarkably, driven by the desire to understand their impact on cognitive performance across the lifespan and improve cognitive function in various populations, including students, healthy adults and the elderly (Cohen Kadosh et al., 2021; Klimova et al., 2020). Caffeine is the most widely consumed psychoactive substance and among the most promising cognitive function enhancers (Cappelletti et al., 2015; James, 2014). Acute caffeine intake is shown to enhance simple cognitive functions such as attention and reaction time but less consistent effects are observed for complex functions such as decision making (McLellan et al., 2016). There are also data showing a positive relationship between habitual caffeine consumption with memory and executive function, but there is limited evidence for an association with simple cognitive functions (Johnson-Kozlow et al., 2002).

Regular caffeine exposure may induce a form of brain preconditioning, with reported alterations of brain functional connectivity, brain metabolism and levels of adenosine and adenosine receptors in the adult brain (Lopes et al., 2023). Brain imaging studies have shown variability in brain regions related to vision, motor and emotional processing between low and high-caffeine consumers (Laurienti et al., 2002). Such changes have been replicated in non-caffeine consumers after a single coffee intake, suggesting possible causality between caffeine intake and altered patterns of neuronal networks (Magalhães et al., 2021), highlighting the importance of accounting for habitual caffeine consumption when examining caffeine acute effects. Studies further suggest that caffeine, particularly in combination with glucose, may enhance attentional efficiency through neural mechanisms that increase processing efficiency in specific regions such as the parietal and prefrontal cortex (Adan and Serra-Grabulosa, 2010; Serra-Grabulosa et al., 2010).

Inconsistencies in caffeine research on cognition are often due to variable cognitive assessment methods, participant selection (habitual consumers vs caffeine-naïve individuals), differences in the form, dosage and mode of caffeine intake (with or without sugar) and potential misclassification of habitual caffeine consumption (Cappelletti et al., 2015; James, 2014). As Nutrigenetics Science has advanced, some of the variability in study results has been attributed to common genetic variations, specifically Single Nucleotide Polymorphisms (SNPs), which are associated with caffeine metabolism and response (Nehlig, 2018). Research suggests that variants in the Adenosine Receptor A2a (*ADORA2A*) gene may influence an individual’s response to caffeine, leading to caffeine-induced anxiety and insomnia (Alsene et al., 2003; Childs et al., 2008). Moreover, variants in the Cytochrome P450 1A2 (*CYP1A2*) and Aryl Hydrocarbon Receptor (*AHR*) genes are associated with variability in caffeine metabolism (Granados et al., 2022; Larigot et al., 2018).

Nevertheless, a recent systematic review demonstrated that genetics studies on habitual caffeine intake and cognitive performance are limited and show mixed results, with some suggesting a relationship between caffeine intake and cognitive abilities in certain genotype groups and others reporting no significant gene × caffeine interactions (Kapellou et al., 2023). Notably, most genetic studies on caffeine and cognition focus on a single variant, such as those limited to caffeine metabolism or response (Baur et al., 2021; Carswell et al., 2020; Casiglia et al., 2017; Skeiky et al., 2020), without considering genetic variants associated with factors affecting cognitive performance, such as sleep. Everyday cognitive performance is influenced by various factors including environmental conditions, sounds, diet and sleep (Cunningham et al., 2018; Lamport et al., 2014; Nemati et al., 2019). Therefore, research should account for these intrapersonal and contextual factors (Verhagen et al., 2019). Additionally, past studies often focus on specific cognitive domains with one-time assessments, whereas repetitive tasks in real-life settings may more accurately capture daily cognitive function (Bouvard et al., 2018).

The aim of the present study was to investigate the interactions between genetics and habitual caffeine consumption on cognitive performance in all key domains of cognition that have been previously linked with caffeine, namely social and emotional cognition, memory, attention and executive function in healthy individuals in real-life conditions.

## Materials and methods

### Participants

A sample size of 131 was calculated using G*Power 3.1 (Faul et al., 2009) for a medium effect size (*f*^2^ = 0.15) at 80% power and an alpha level of 5% for 13 influencers of cognitive function: (1) age, (2) sex, (3) body mass index (BMI), (4) habitual caffeine intake, (5) genetic variants in genes involved in caffeine metabolism (*AHR* rs6968554 and *CYP1A2* rs2472297) and response (*ADORA2A* rs5751876), (6) physical activity, (7) level of education, (8) subjective sleep quality and (9) subjective sleepiness prior to tasks, (10) tobacco, (11) alcohol use, (12) *ADA* rs73598374 and (13) *APOE* ε4−/ε4+ genes (Campos et al., 2016; Erickson et al., 2019; Harada et al., 2013; McLellan et al., 2016; Michaud et al., 2018; Wang et al., 2022). The ADA rs73598374 and APOE ε4+/ε4− were used as proxies of sleep quality and risk of cognitive impairment, respectively (Gharbi-Meliani et al., 2021; Tartar et al., 2021).

Participants were recruited via email, word of mouth and social media. Data collection was completed remotely while genotyping and statistical analyses were completed at St Mary’s University Twickenham. Adult males and females in the UK with no neurocognitive disorders or vision impairments were eligible. Exclusion criteria included medication that may alter *CYP1A2* enzyme activity, including oral contraceptives, exogenous hormones, Selective Serotonin Reuptake Inhibitors and quinolone antibiotics (Arnaud, 2011; Casiglia et al., 2017; Grzegorzewski et al., 2021), as well as pregnant or lactating women. Participant involvement lasted 3 days.

### Baseline questionnaire

**Figure 1.**
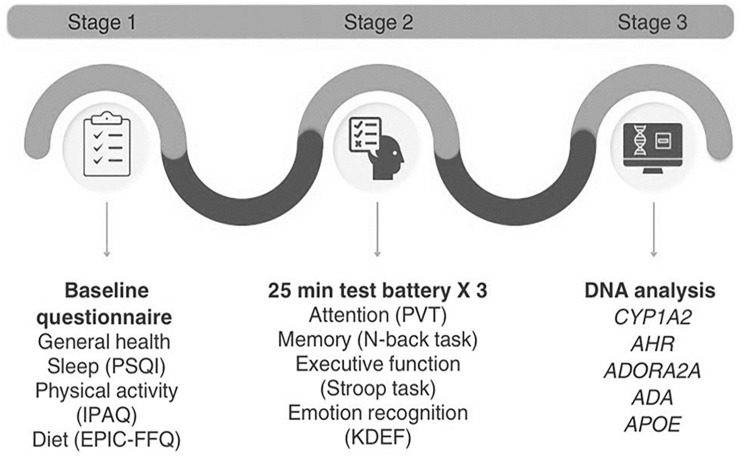
Schematic representation of participant involvement across the three stages of the study. PSQI: Pittsburgh sleep quality index; IPAQ: international physical activity questionnaire; EPIC-FFQ: EPIC food frequency questionnaire; PVT: psychomotor vigilance task; KDEF: Karolinska directed emotional faces.

The first part of the study included a baseline questionnaire consisting of sociodemographic, health and lifestyle questions (Figure 1).

Sociodemographic questions included sex, age, ethnic origin, education level (scored 1–7 for higher qualifications), medical history, medications, and tobacco use. Physical activity was assessed using the short International Physical Activity Questionnaire (IPAQ), classifying activity levels as ‘low’, ‘moderate’ or ‘high’ (Hallal and Victora, 2004). Sleep quality was measured using the Pittsburgh Sleep Quality Index (PSQI), with scores of 0–4 indicating ‘good’ sleep and 5–21 indicating ‘poor’ sleep (Fabbri et al., 2021).

Participants completed the EPIC-Norfolk Food Frequency Questionnaire (FFQ) to assess habitual caffeine and alcohol intake (Mulligan et al., 2014). Caffeine intake was calculated from 11 common foods and drinks, including tea, coffee, chocolate and soft drinks, using EPIC database portion sizes and published caffeine content data (Fitt et al., 2013; Malczyk et al., 2021).

### Cognitive test battery

In the second part of the study, participants completed a cognitive test battery hosted by the Gorilla Experiment Builder (Anwyl-Irvine et al., 2020) on three separate days within a period of 2 weeks. The battery comprised four tasks covering (in order of appearance) social and emotional cognition, memory, attention and executive function. Participants were instructed to complete the tasks on the same device in a quiet room and at least 5 h after consuming any source of caffeine or alcohol, hypothesising that individuals would be neither under the acute effects of these substances nor under caffeine abstinence when the test battery was performed (Casiglia et al., 2017). Subjective sleepiness before each test session was assessed using the Karolinska Sleepiness Scale (KSS), a validated 9-point Likert scale ranging from ‘1’ (extremely alert) to ‘9’ (extremely sleepy, fighting sleep) (Kaida et al., 2006). The time of test completion was recorded for all tasks.

#### Social and emotional cognition

Participants completed a facial expression recognition task using 48 images of faces showing six basic emotions (anger, fear, sadness, happiness, disgust and surprise) from the Karolinska Directed Emotional Face System (Lundqvist et al., 1998). Images were displayed for 1 s and participants selected the emotion for each face (3500 ms interstimulus interval). Mean reaction time (RT) for correct responses over 3 days was used in analyses.

#### Memory

Memory was assessed using the 1-, 2-, and 3-back letter tasks (Owen et al., 2005). Each task involved 20 targets and 40 non-targets, where participants pressed ‘Yes’ for targets and ‘No’ for non-targets (3500 ms interstimulus interval). The three-day mean RT for correct answers was analysed, excluding responses <100 ms.

#### Attention

The Psychomotor Vigilance Task (PVT) measured the domain of attention (Lim and Dinges, 2008). Participants pressed a button when a red dot appeared on a scrolling time counter. The interstimulus interval varied from 2 to 10 s over 100 trials. RTs < 100 ms and >500 ms were excluded, and the 3-day mean RT was used.

#### Executive function

The Stroop colour and word task assessed executive function. Participants named the colour of words in two subsets: neutral (black words) and incongruent (words in mismatched colours). This task is based on the principle that reading is a more automatic response than colour naming, leading to interference (Stroop asynchrony) (Scarpina and Tagini, 2017). The 3-day mean Stroop effect was calculated as the difference between mean RTs for the coloured and non-coloured sets.

#### Global cognition

A composite global cognition score was computed by averaging z-scores from the four cognitive tasks, with all RTs converted into response speed (1/RT) to standardise scoring. This provided a more comprehensive representation of cognitive function (Zhou et al., 2018).

### Genotyping

In the final part of the study, participants with complete data from the baseline questionnaire and test battery were provided a buccal swab (RapiDri™ Swab kit, Isohelix, Kent, UK) for DNA analysis. Swabs were mailed home with a pre-paid return envelope and sampling instructions.

Laboratory analyses followed approved protocols by the St Mary’s Ethics Committee. DNA was extracted using the PSP^®^ SalivaGene 17 DNA Kit (STRATEC Molecular, Berlin) following the manufacturer protocol. Genotyping for *AHR* rs6968554, *CYP1A2* rs2472297, *ADORA2A* rs5751876, *ADA* rs73598374, and *APOE* ε4+/ε4− (rs429358 and rs7412) was performed using TaqMan^®^ SNP genotyping assays (ThermoFisher) and a StepOnePlus thermocycler (Applied Biosystems, CA, USA), with individual samples accepted if quality was >98%.

### Statistical analysis

Statistical analysis was conducted using IBM SPSS Statistics 28.0 (IBM, Chicago, IL). Data are presented as means ± standard deviation (SD) or medians ± interquartile range (IQR). Participants were grouped based on the level of habitual caffeine intake into low (0–50 mg/day), moderate (51–300 mg/day), and high (>300 mg/day) caffeine consumers (Erblang et al., 2019). Group differences were assessed using one-way ANOVA or Kruskal–Wallis *H* test. Categorical differences were analysed with Chi-square or Fisher’s exact test.

Deviations from Hardy Weinberg Equilibrium (HWE) for each SNP were performed using the *χ*^2^ goodness-of-fit test. For caffeine metabolism, an unweighted score (0–4) was calculated based on alleles associated with faster metabolism in *CYP1A2* rs2472297 and *AHR* rs6968554 (Cornelis et al., 2016). Scores of 0–1 indicated ‘slow’ and 2–4 indicated ‘fast’ caffeine metabolism. For caffeine sensitivity, *ADORA2A* genotypes were grouped into ‘sensitive’ (TT group) and ‘non-sensitive’ (C allele carriers) (Alsene et al., 2003; Childs et al., 2008; Rogers et al., 2010). Finally, an overall genetic caffeine score (0–6) was constructed by combining caffeine metabolism and sensitivity alleles, with higher scores indicating faster caffeine metabolism and lower caffeine sensitivity. The *APO* ε4+/ε4− genotype was determined by the combinations of genotypes at rs429358 and rs7412 (Fernández-de-las-Peñas et al., 2023). The *ADA* rs73598374 and *APO* ε4+/ε4− were used in the analyses separately, as proxies of sleep quality and risk of cognitive impairment, respectively.

Genotype groups were compared using independent samples *t*-test or Mann–Whitney *U* test. Two-way ANOVA with Bonferroni adjustments assessed interactions between genetic scores and caffeine intake on cognition. Multiple regression was used to analyse the relationship between caffeine, genetics and cognition, adjusting for potential influencers (age, sex, BMI, education, sleep quality, tobacco/alcohol use, *ADA* rs73598374 and *APOE* ε4+/ε4−). Statistical significance was assumed at the 5% level.

## Results

### Participant characteristics across levels of caffeine intake

A total of 129 participants aged 23–64 years (94% Caucasian/white) were included in the study (Table 1). Most participants had moderate PAL (74%) and a normal BMI of 18.5–24.9 kg/m^2^ (57%). All participants consumed alcohol within safe limits (<100 g/week; Conigrave et al., 2021). Genotype frequencies for all SNPs did not deviate from HWE (*p*_all_ > 0.05).

**Table 1.** Participant characteristics by total sample and level of habitual caffeine consumption.

Characteristic	All (*N* = 129)	*L* (*N* = 19)	*M* (*N* = 85)	*H* (*N* = 25)	*p* *
Age^b^, years	35.0 ± 15.5	33.0 ± 9.0	34.0 ± 15.5	37.0 ± 13.5	0.261
Gender, F/M (%)	90 (69.8)/39 (30.2)	14 (73.7)/5 (26.3)	62 (72.9)/23 (27.1)	14 (56.0)/11 (44.0)	0.248
BMI^b^ (kg/m^2^)	23.9 ± 6.5	25.5 ± 9.7	23.7 ± 5.2	24.2 ± 9.1	0.373
Ethnicity, white/non-white (%)	121 (93.8)/8 (6.2)	16 (84.2)/3 (15.8)	80 (94.1)/5 (5.9)	25 (100.0)/0 (0.0)	0.106
Education level^b^	6.0 ± 1.0	5.0 ± 1.0	6.0 ± 1.0	5.0 ± 1.0	0.427
PAL, *L*/*M*/*H* (%)	12 (9.8)/94 (73.8)/23 (16.4)	1 (5.3)/15 (78.9)/3 (15.8)	7 (8.2)/62 (72.9)/16 (18.8)	4 (16.0)/17 (68.0)/4 (16.0)	0.789
Smoking status, no/yes (%)	110 (85.3)/19 (14.7)	19 (100.0)/0 (0.0)	71 (83.5)/14 (16.5)	20 (80.0)/5 (20.0)	0.115
Sleep quality, poor/good (%)	82 (63.6)/47 (36.4)	11 (57.9)/8 (42.1)	55 (64.7)/30 (35.3)	16 (64.0)/9 (36.0)	0.855
PSQI^b^	5.0 ± 3.0	5.0 ± 3.0	5.0 ± 4.0	6.0 ± 4.0	0.283
KSS^b^	5.0 ± 3.0	5.0 ± 4.0	5.0 ± 3.0	5.0 ± 1.0	0.046
Exam time, before 12 pm/after 12 pm (%)	19 (14.7)/110 (85.3)	4 (21.1)/15 (78.9)	11 (12.9)/74 (87.1)	4 (16.0)/21 (84.0)	0.576
Alcohol intake^b^ (g/week)	14.5 ± 33.0	8.9 ± 12.2	21.8 ± 37.1	5.6 ± 27.1	0.017
Caffeine intake^b^ (mg/day)	184.5 ± 153.9	11.6 ± 22.1	176.9 ± 120.8	382.8 ± 116.4	<0.001
*AHR* genotype (%)					
AA	17 (13.2)	2 (10.5)	13 (15.3)	2 (8.0)	0.927
AG	70 (54.3)	11 (57.9)	44 (51.8)	15 (60.0)	
GG	42 (32.6)	6 (31.6)	28 (32.9)	8 (32.0)	
*CYP1A2* genotype (%)					
CC	102 (79.1)	17 (89.5)	66 (77.6)	19 (70.4)	0.641
CT	23 (17.8)	2 (10.5)	15 (17.6)	8 (29.6)	
TT	4 (3.1)	0 (0.0)	4 (4.8)	0 (0.0)	
*ADORA2A* genotype, sensitive/non-sensitive (%)	30 (23.3)/99 (76.7)	5 (26.3)/14 (73.7)	16 (18.8)/69 (81.2)	9 (36.0)/16 (64.0)	0.191
*ADA* genotype, GG/AG (%)	126 (97.7)/3 (2.3)	19 (100.0)/0 (0.0)	83 (97.6)/2 (2.4)	24 (96.0)/1 (4.0)	0.717
*APOE* genotype, ε4 carriers/ε4 noncarriers (%)	29 (22.5)/100 (77.5)	6 (31.6)/13 (68.4)	14 (16.5)/71 (83.5)	9 (36.0)/16 (64.0)	0.060

Low, moderate, and high-caffeine intake: 0–50, 51–300 and >300 mg/day, respectively.

L: low; M: moderate; H: high; PAL: physical activity level; BMI: body mass index; PSQI: Pittsburgh sleep quality index; KSS: Karolinska sleepiness scale.

a Values represent means ± SD.

b Values represent medians ± IQR.

* *p*-values represent differences across the three levels of caffeine intake, assessed by one-way ANOVA, Kruskal–Wallis *H* test or Chi-square test.

Two participants were excluded from attention analyses due to RTs above 500 ms on all 3 days of the experiment, and 12 were excluded from executive function analyses for incorrectly performing the task. This left 117 participants for the global cognition analysis.

Demographics did not differ across caffeine intake groups, while differences were observed in subjective sleepiness, alcohol and caffeine intake (*p*_all_ < 0.05), as assessed by the Kruskal–Wallis test using Dunn’s procedure with a Bonferroni correction for multiple comparisons. Post hoc analysis revealed significantly higher alcohol intake in moderate versus high-caffeine consumers (*p* = 0.026). For subjective sleepiness, post hoc analysis revealed significantly higher subjective sleepiness scores in high (5.0 ± 1.0) compared with low (5.0 ± 4.0) (*p* = 0.046) caffeine intake groups.

### Participant characteristics across genetic groups

In the present sample, MAF for *CYP1A2* rs2472297 (T allele) was 0.12, which is lower than expected for a European population and 0.40 for *AHR* rs6968554 (A allele), which is in line with published data for a population of European descent. The MAF for *ADORA2A* rs5751876 (T allele) was 0.43, which is slightly higher than published data for Europeans (Cunningham et al., 2022).

Participants were grouped into ‘fast’ and ‘slow’ metabolisers using two SNPs (*CYP1A2* rs2472297 and AHR rs6968554) linked to caffeine plasma metabolites (Cornelis et al., 2016). No differences were found between groups except for habitual caffeine consumption, with ‘fast’ metabolisers consuming significantly more caffeine than ‘slow’ metabolisers (*p* = 0.045, Table 2).

**Table 2.** Participant characteristics by genetic caffeine metabolism.

Characteristic	Slow metabolisers (*N* = 71)	Fast metabolisers (*N* = 58)	*p* *
Age^b^, years	36.0 ± 16.0	34.0 ± 12.0	0.264
Gender, F/M (%)	47 (66.2)/24 (33.8)	43 (74.1)/15 (25.9)	0.329
BMI^b^ (kg/m^2^)	27.2 ± 7.9	24.3 ± 4.4	0.549
Ethnicity, white/non-white (%)	65 (91.5)/6 (8.5)	56 (96.6)/2 (3.4)	0.294
Education level^b^	5.0 ± 1.0	6.0 ± 1.0	0.142
PAL, *L*/*M*/*H* (%)	6 (8.5)/49 (69.0)/16 (22.5)	6 (10.3)/45 (77.6)/7 (12.1)	0.300
Smoking status, no/yes (%)	61 (85.9)/10 (14.1)	49 (84.5)/9 (15.5)	0.819
Sleep quality, poor/good (%)	41 (57.7)/30 (42.3)	41 (70.7)/17 (29.3)	0.129
PSQI^b^	5.0 ± 4.0	6.0 ± 3.3	0.088
KSS^b^	5.0 ± 3.0	5.0 ± 2.0	0.865
Exam time, before 12 pm/after 12 pm (%)	13 (18.3)/58 (81.7)	6 (10.3)/52 (89.7)	0.204
Alcohol intake^b^ (g/week)	16.4 ± 34.1	12.8 ± 33.3	0.972
Caffeine intake^b^ (mg/day)	124.0 ± 135.1	212.3 ± 203.4	0.045
*ADORA2A* genotype, sensitive/non-sensitive (%)	12 (16.9)/59 (83.1)	18 (31.0)/40 (69.0)	0.059
*ADA* genotype, GG/AG (%)	71 (100.0)/0 (0.0)	55 (94.8)/3 (5.2)	0.088
*APOE* genotype, ε4 carriers/ε4 noncarriers (%)	16 (22.5)/55 (77.5)	13 (22.4)/45 (77.6)	0.987

Participants are categorised according to *AHR* + *CYP1A2* genotypes (‘slow’ or ‘fast’ metabolisers).

BMI: body mass index; PAL: physical activity level; L: low; M: moderate; H: high; PSQI: Pittsburgh sleep quality index; KSS: Karolinska sleepiness scale.

a Values represent means ± SD.

b Values represent medians ± IQR.

* *p*-values represent differences between genotype groups, assessed by independent samples *t*-test, Mann–Whitney *U* test or Chi-square test.

The *ADORA2A* SNP, previously associated with caffeine-induced anxiety (Alsene et al., 2003; Childs et al., 2008) was used to group participants among caffeine ‘sensitive’ and ‘non-sensitive’ individuals. Using this classification, 30 (24%) participants were classified as ‘sensitive’ and 99 (76%) were classified as ‘non-sensitive’ individuals. Participant characteristics were not different between groups, as assessed by the Mann–Whitney *U* test (results not shown).

### Cognitive performance

Multiple regressions (*n* = 5) were run to investigate the associations between cognitive performance and factors including age, sex, BMI, habitual caffeine intake, overall caffeine genetic score, PAL, education, subjective sleep quality, subjective sleepiness prior to tasks, tobacco and alcohol use, genetic sleep quality and genetic risk for cognitive decline.

The proportion of variance in all models was of trivial effect based on Cohen (1988), with the model of social and emotional cognition explaining 10% of variance, the model of memory explaining 16.6% of variance, the model of attention accounting for 5.3% of variance, the model of executive function accounting for 10.1% of variance and the model of global cognition explaining 15.7% of the variation in performance. The factors associated with cognitive function included age (social and emotional cognition, memory, attention and global cognition) and BMI (executive function). Neither habitual caffeine intake nor genetics were associated with cognitive function in any of the measures.

### Habitual caffeine intake × genetics on cognition

**Figure 2.**
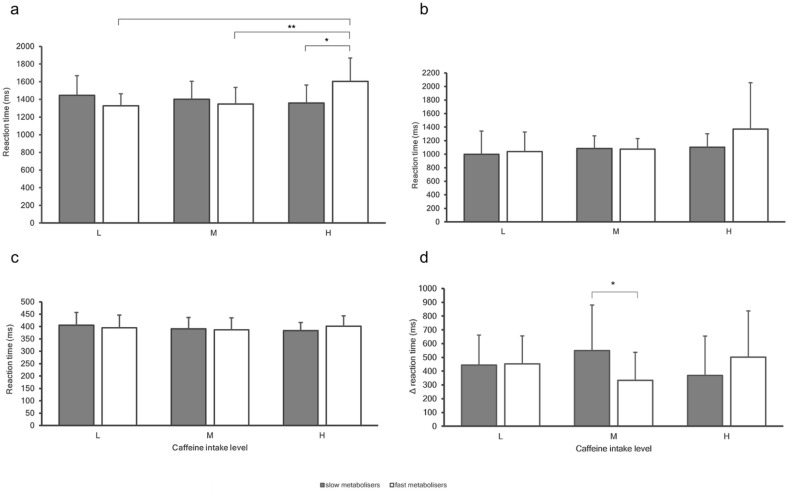
Task mean reaction times for ‘slow’ (grey bars) and ‘fast’ (white bars) metabolisers for levels of caffeine intake (low vs moderate vs high) in (a) emotion recognition, (b) memory, (c) attention and (d) executive function. Error bars indicate standard deviations. *p < 0.05. **p < 0.01.

**Figure 3.**
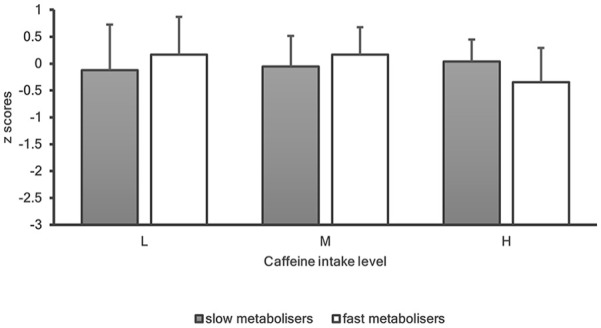
Overall cognitive performance *z*-scores for ‘slow’ (grey bars) and ‘fast’ (white bars) metabolisers for levels of caffeine intake (low vs moderate vs high). Error bars indicate standard deviations.

The possible interactions between genetic caffeine metabolism and levels of caffeine intake on indices of cognition were investigated and results are shown in Figures 2 and 3.

Two-way ANOVAs were conducted to examine the associations of habitual caffeine intake and genetic caffeine metabolism on cognitive performance. All pairwise comparisons were run for each simple main effect with a reported 95% confidence interval (CI). All *p*-values were Bonferroni-adjusted within each simple main effect and were accepted at the *p* < 0.025 level for two simple main effects and *p* < 0.017 for three simple main effects.

There was a statistically significant interaction between levels of habitual caffeine intake and genetic caffeine metabolism for emotion recognition, *F*_2, 123_ = 5.848, *p* = 0.004 and executive function, *F*_2, 109_ = 3.690, *p* = 0.028, but not for any other indices of cognition. A statistically significant difference was found in emotion recognition performance between ‘slow’ and ‘fast’ metabolisers in high-caffeine consumers, *F*_1, 123_ = 8.835, *p* = 0.004. Within high-caffeine consumers, mean emotion recognition RTs for ‘fast’ metabolisers were 246.3 (95% CI: 82.290–410.371) ms higher compared with ‘slow’ metabolisers (1604.5 ± 263.8 ms vs 1358.2 ± 204.5 ms, respectively, *p* = 0.004). No differences were observed between low and moderate caffeine consumers. There was also a statistically significant difference in emotion recognition performance among levels of caffeine intake in ‘fast’ metabolisers, *F*_2, 123_ = 8.512, *p* < 0.001. Within ‘fast’ metabolisers, mean emotion recognition RTs for high-caffeine consumers were 276.9 (95% CI: 45.797–507.992) ms higher compared with low (1604.5 ± 263.8 ms vs 1327.6 ± 136.7 ms, respectively, *p* = 0.013) and 256.8 (95% CI: 100.139–413.431) ms higher compared with moderate (1604.5 ± 263.8 ms vs 1347.7 ± 187.6 ms, respectively, *p* < 0.001) caffeine consumers. No differences were observed within ‘slow’ metabolisers.

There was a statistically significant difference in executive function performance between ‘slow’ and ‘fast’ metabolisers in moderate caffeine consumers, *F*_1, 109_ = 10.583, *p* = 0.002. Within moderate caffeine consumers, the mean executive function ΔRTs for ‘slow’ metabolisers was 215.9 (95% CI: 84.374–347.479) ms higher compared with ‘fast’ metabolisers (549.3 ± 330.9 ms vs 333.4 ± 203.6 ms, respectively, *p* = 0.002). No differences were observed between low and high-caffeine consumers.

No comparisons were performed between caffeine ‘sensitive’ and ‘non-sensitive’ individuals based on the level of habitual caffeine intake since there were only three participants in the ‘sensitive’ – low-caffeine intake group.

## Discussion

The purpose of the present study was to investigate the interactions between genetics and habitual caffeine consumption on cognitive performance in four key domains of cognition, namely social and emotional cognition, memory, attention and executive function in healthy individuals in real-life conditions and on three separate occasions. The present study showed that habitual caffeine intake was higher in ‘fast’ compared with ‘slow’ metabolisers. Moreover, ‘slow’ metabolisers performed better than ‘fast’ metabolisers in emotion recognition among high-caffeine consumers. On the contrary, ‘fast’ metabolisers performed better than ‘slow’ metabolisers in the executive function domain, but only within moderate caffeine consumers. The findings are discussed in detail below.

### Habitual caffeine intake

Genetics are shown to be involved in individual variability in caffeine consumption, both at the pharmacodynamic and pharmacokinetic levels (Yang et al., 2010). This study found a significant difference in caffeine intake between genetic metabolism groups, with ‘fast’ metabolisers consuming more than ‘slow’ metabolisers (*p* = 0.045). The SNPs employed in the current analysis as proxies for caffeine metabolism have been shown to be associated with habitual caffeine consumption (Cornelis et al., 2011, 2015), supporting the hypothesis that individuals self-regulate their caffeine intake for optimal level of arousal (Zhou et al., 2010). Therefore, ‘fast’ caffeine metabolisers may need more caffeine to avoid symptoms of abstinence and withdrawal.

On the contrary, this study did not replicate previous findings of an association between the *ADORA2A* gene and habitual caffeine intake. It has been reported that carriers of the TT genotype in rs5751876 are more likely to consume less caffeine than carriers of the C allele (Cornelis et al., 2007), suggesting a role of caffeine in the regulation of anxiety (Alsene et al., 2003). However, the present study had a smaller sample size compared with the study by Cornelis et al. (2007) and, despite the apparent plausibility of this association, this finding has not been replicated in GWAS of habitual caffeine consumption.

### Cognitive performance

The current analysis supports a domain-specific association between habitual caffeine consumption and cognitive function. Age and BMI were the only factors associated with performance across various cognitive indices. The results are discussed below by cognitive domain.

#### Social and emotional cognition

Social and emotional cognition refers to all mental processes underlying one’s ability to understand the behaviours of others, as a requirement for communication and social interactions (Pinkham et al., 2014). The present study showed that age is negatively associated with performance in the domain of social and emotional cognition, consistent with previous findings (Adólfsdóttir et al., 2017; Tremblay et al., 2016).

Moreover, a statistically significant difference in emotion recognition between ‘slow’ and ‘fast’ metabolisers among high-caffeine consumers is reported. Specifically, ‘slow’ metabolisers had a higher performance in emotion recognition compared with ‘fast’ metabolisers, but only among high-caffeine consumers (*p* = 0.004). Moreover, among ‘fast’ metabolisers, high-caffeine consumers had a lower performance in emotion recognition compared with those consuming low and moderate levels of caffeine.

Lower performance among ‘fast’ metabolisers who consume high levels of caffeine may be due to withdrawal symptoms. Participants abstained from caffeine for at least 5 h before testing, which means that habitual caffeine consumers would be neither under the acute effects of caffeine nor in abstinence when cognitive tasks would be performed (Casiglia et al., 2017). A typical overnight caffeine abstinence results in substantial elimination of systemic caffeine by early morning; thus, upon awakening, caffeine consumers have entered the early stages of caffeine withdrawal (Juliano and Griffiths, 2004). Although test times were similar across groups, ‘fast’ metabolisers might have experienced stronger withdrawal, especially if tested in the morning before their usual caffeine intake, potentially affecting their performance.

Finally, high-caffeine consumers reported greater sleepiness before tasks compared to low consumers, aligning with previous research linking heavier caffeine use to higher daytime sleepiness (Chaudhary et al., 2016). This sleepiness may have contributed to their lower performance, as levels of alertness may impact cognitive function (Fortier-Brochu et al., 2012).

This study is the first to explore the associations between caffeine and the domain of social cognition. Despite the documented effects of caffeine on emotional arousal (Giles et al., 2018), its association with emotion recognition is unclear and warrants further study.

#### Memory

In the memory domain, higher age was linked to lower performance, consistent with previous findings (Adólfsdóttir et al., 2017; Salthouse, 2012). No significant gene x caffeine interactions were observed, aligning with the UK Biobank studies using the same genetic score for caffeine metabolism (Cornelis et al., 2020a, 2020b). However, despite SNP selection, these studies assessed memory and habitual caffeine intake differently. The present study used the n-back task to assess working memory, while the UK Biobank studies focused on prospective and episodic memory. Additionally, this study measured caffeine intake from all dietary sources, whereas the UK Biobank focused on recent coffee or tea intake within the hour preceding cognitive assessments, or habitual caffeine intake in cups of coffee or tea per day.

#### Attention

Performance in the domain of attention was associated with age; the higher the age, the lower performance in attention, as previously shown (Adólfsdóttir et al., 2017; Tremblay et al., 2016). No significant gene × caffeine interactions were observed for the domain of attention. This is also in line with the investigations from the UK Biobank, which failed to find any gene × caffeine interactions for the test of vigilance. Nevertheless, the studies used different cognitive tasks to assess vigilant attention (PVT vs symbol matching). Therefore, the implications of the different methodologies between the studies, as mentioned for the domain of memory, need to be considered.

#### Executive function

Higher BMI was associated with lower performance in executive function, which is in line with the existing literature (Lokken et al., 2009; Nilsson and Nilsson, 2009).

A statistically significant difference was observed in executive function performance between ‘slow’ and ‘fast’ metabolisers among moderate caffeine consumers. Among moderate caffeine consumers, ‘fast’ metabolisers had a higher performance in executive function compared with ‘slow’ metabolisers (*p* = 0.002). This suggests a dose-specific association between caffeine intake and executive function, where ‘fast’ metabolisers perform better than ‘slow’ metabolisers, but only with moderate caffeine intake. Moderate doses may provide cognitive benefits for ‘fast’ metabolisers without the negative effects of withdrawal, anxiety, restlessness, or sleep disturbance (Alsene et al., 2003; Childs et al., 2008). In contrast, it is possible that in ‘slow’ metabolisers, the steady-state plasma/brain caffeine concentration may have been sufficient to discourage caffeine intake (Cornelis et al., 2015), yet not enough to improve executive function.

Executive function has been examined in three prior genetic studies on caffeine. Two studies from the UK Biobank reported no significant associations when using a verbal-numerical reasoning test (Cornelis et al., 2020a, 2020b). In contrast, another study found that higher caffeine intake was linked to greater abstraction scores, but only among ‘slow’ metabolisers (Casiglia et al., 2017). Variations in tasks, testing conditions (laboratory vs at-home), the SNPs used as proxies for genetic caffeine metabolism and methods for measuring habitual caffeine intake contribute to the differing results. Additionally, it is important to note that Casiglia et al. (2017) required participants to abstain from caffeine overnight, which may have influenced findings due to withdrawal symptoms.

#### Global cognition

This was the first genetics study on caffeine and cognition to employ a global cognitive function score. Although age was negatively associated with global cognitive function, no significant gene × caffeine associations were found. Considering the results in the present sample, two questions may arise: the differential results between emotion recognition and executive function and the lack of significant gene x caffeine interactions for the domains of attention, memory and global cognition.

In addition to the nature of cognitive domains (simple vs complex), task order may have influenced results, as the emotion recognition task was administered first, potentially affecting validity. Indeed, mental fatigue can lead to a decline in performance over time, especially in cognitively demanding tasks (Borbély et al., 2016; Caldwell et al., 2019). This is underscored by the number of participants excluded for not following instructions in the executive function task. Although the test duration may not have induced cognitive fatigue (Dallaway et al., 2022), as time on task increases, exerting effort declines, leading to reductions in performance (Müller and Apps, 2019). In this sample, this is especially important for high-caffeine consumers, who presented lower baseline alertness compared with the other caffeine intake groups.

In summary, the findings suggest that the test battery may have lost sensitivity for certain tasks, impacting the detection of associations across all domains. As comparable cognitive tests are limited, caution should be applied in interpreting findings, and replication is warranted.

### Strengths and limitations

This is the first genetic association study on caffeine examining all four key domains of cognition assessing cognitive performance remotely on three separate instances to account for common day-to-day intra-individual variations in performance (von Stumm, 2016) to provide a more appropriate estimate of everyday cognition. Recent findings also support that cognitive tasks performed in naturalistic settings (e.g., at home or at work) provide measurements that are comparable in reliability to assessments made in controlled laboratory environments (Sliwinski et al., 2018).

Nevertheless, this study is not free of limitations. Firstly, because of participant data exclusion, the study was not appropriately powered and this may explain the lack of significance in some cognitive domains. Additionally, the caffeine groups were imbalanced, with only seven participants in the low-caffeine ‘fast’ metaboliser group, potentially impacting the results and highlighting the need for further research. Moreover, significant variations among caffeine intake groups, such as subjective sleepiness prior to tasks and alcohol consumption, as well as non-significant differences in tobacco use, could have influenced cognitive outcomes and should be considered when interpreting the results.

## Conclusions

This study confirms that the association between caffeine and cognition is domain-specific, with social and emotional cognition and executive linked to habitual intake. It also replicates findings that ‘fast’ metabolisers consume more caffeine, likely to achieve psychostimulant effects, with different caffeine-cognition associations due to more profound withdrawal symptoms in ‘fast’ metabolisers. Variations in brain caffeine and paraxanthine levels may explain differences in mental fatigue and engagement. While the results align with existing literature, inconsistent cognitive assessment methods limit their broader applicability. Larger studies in naturalistic settings are needed to confirm these findings and explore gene x caffeine interactions in brain functions.
